# Structural modeling of antibody variable regions using deep learning—progress and perspectives on drug discovery

**DOI:** 10.3389/fmolb.2023.1214424

**Published:** 2023-07-07

**Authors:** Igor Jaszczyszyn, Weronika Bielska, Tomasz Gawlowski, Pawel Dudzic, Tadeusz Satława, Jarosław Kończak, Wiktoria Wilman, Bartosz Janusz, Sonia Wróbel, Dawid Chomicz, Jacob D. Galson, Jinwoo Leem, Sebastian Kelm, Konrad Krawczyk

**Affiliations:** ^1^ NaturalAntibody, Kraków, Poland; ^2^ Medical University of Warsaw, Warsaw, Poland; ^3^ Medical University of Lodz, Lodz, Poland; ^4^ Alchemab Therapeutics Ltd., London, United Kingdom; ^5^ UCB, Slough, United Kingdom

**Keywords:** deep learning, structural modeling, drug discovery, antibody therapeutics, antibody structure prediction

## Abstract

AlphaFold2 has hallmarked a generational improvement in protein structure prediction. In particular, advances in antibody structure prediction have provided a highly translatable impact on drug discovery. Though AlphaFold2 laid the groundwork for all proteins, antibody-specific applications require adjustments tailored to these molecules, which has resulted in a handful of deep learning antibody structure predictors. Herein, we review the recent advances in antibody structure prediction and relate them to their role in advancing biologics discovery.

## 1 Introduction

Antibodies are the largest class of biotherapeutics, with more than 100 approved molecules (Kaplon et al., 2023). The antibody drug market is rapidly growing, and it is predicted to reach approximately $300 billion by 2025 (Lu et al., 2020). As a result, there is much interest in streamlining antibody discovery methods by tapping into recent computational advances in deep learning.

One of the most striking computational advances has taken place in structure prediction, with the development of tools such as AlphaFold2 (Jumper et al., 2021). For antibodies, the determination of the proper antibody structure is key to many downstream drug discovery tasks, such as developability annotation (Raybould et al., 2019) or antibody–antigen docking (Krawczyk et al., 2014; Schneider et al., 2021). Though AlphaFold2 works well for general proteins, it falls short on the specific case of antibodies (Ruffolo et al., 2022a; Abanades et al., 2022b; Cohen et al., 2022), prompting the development of antibody-specific modeling protocols.

In this review, we describe the methods which contribute to the improvement of computational structure modeling for antibodies and provide context to the role they play in designing antibody-based therapeutics.

## 2 Antibody structure in the context of 3D modeling

**FIGURE 1:**
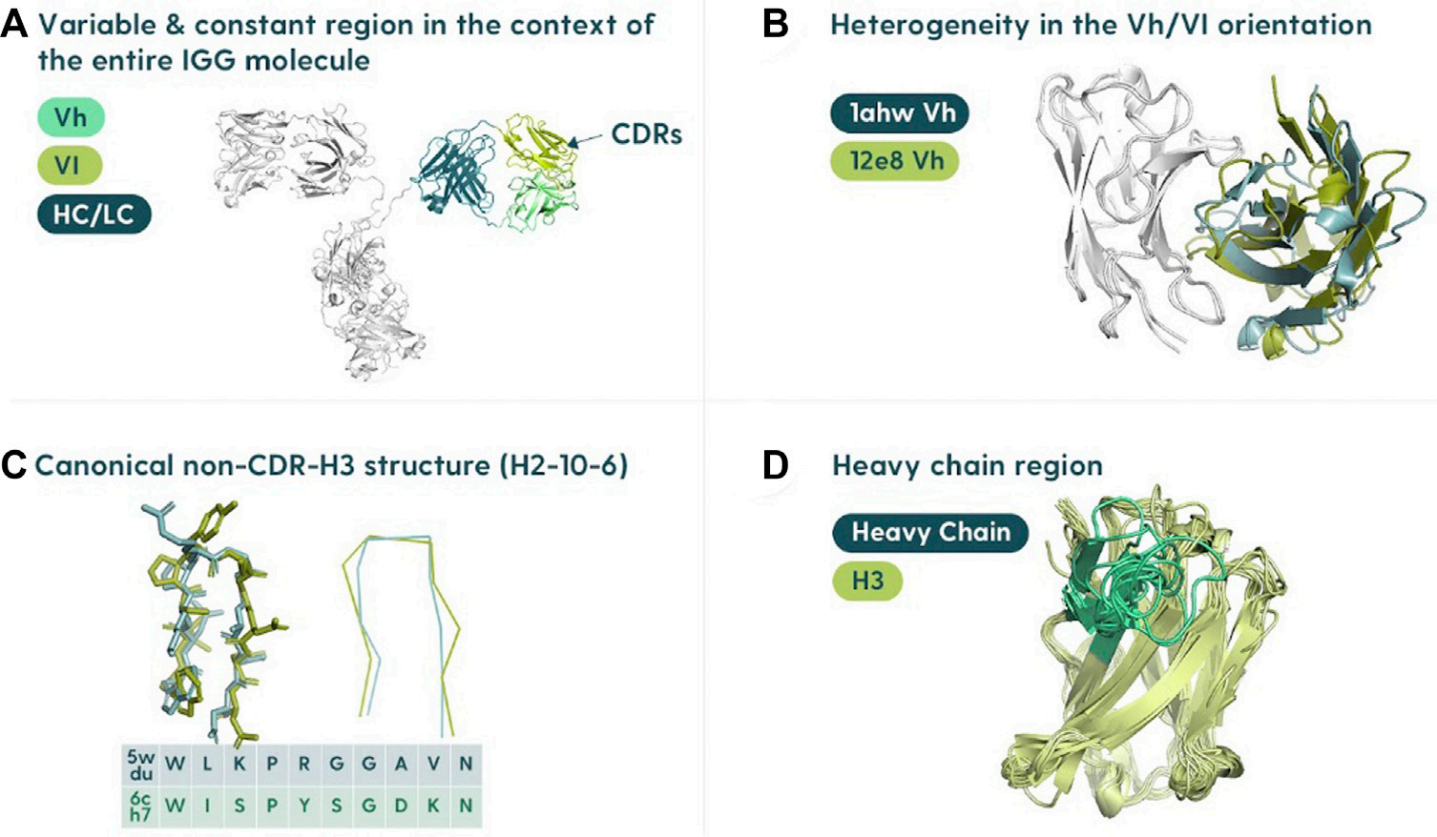
Specifics of the antibody structure in the context of modeling. **(A)** Variable region in the context of the entire antibody structure. The antibody binding site is located in the variable region composed of the variable heavy (Vh) and variable light (Vl) polypeptide chains associated with the constant portions (HC/LC). **(B)** Heavy/light chain orientation. The orientation of the Vh and Vl is not constant, and differing angles can affect the shape of the binding site. **(C)** Canonical structures of CDRs. Most of the binding residues (the paratope) are found in the complementarity-determining regions (CDRs). There are three CDRs on each of the heavy and light chains. All the CDRs except the CDR-H3 cluster into a set of “canonical shapes” depending on residues in key positions. **(D)** Heterogeneity of CDR-H3. CDR-H3 is not only the most variable of the regions but also usually the most important for antigen binding.

Antibody structure prediction is primarily focused on the variable domains of the heavy chain (Vh) and the light chain (Vl) (Figure 1A). Each domain is relatively small, comprising ∼110 residues each. There are two major hurdles within the overall antibody structure prediction problem: determining the relative orientation of the two domains (Figure 1B) and predicting the complementarity-determining region (CDR) loop structures. The two domains can be juxtaposed differently, which affects the overall shape of the antibody binding site. For this reason, orientating the multimer of the heavy and light chains is crucial (Dunbar et al., 2013; Bujotzek et al., 2015).

The CDR prediction problem can be further subdivided into classifying the “canonical” CDRs (CDR-L1, CDR-L2, CDR-L3, CDRH1, and CDR-H2) or modeling the CDR-H3. The canonical CDRs have reasonably conserved folds (Nowak et al., 2016; Kelow et al., 2022) (Figure 1C). The latter problem is arguably the most difficult and critical, as the CDR-H3 is the most variable (Figure 1D), and also plays the major role in binding (Marks and Deane, 2017; Regep et al., 2017; Ruffolo et al., 2020; Abanades et al., 2022a).

There is a diversity of methods to approach any of these sub-problems individually, or predicting the entire multimeric gamut of variable domains. However, attention is often focused around CDR-H3 prediction accuracy given its central role in binding and function. Compilation of the available antibody structure prediction methods that leverage recent advances in machine learning are listed in Table 1.

**TABLE 1:** Compilation of the available antibody structure prediction methods that leverage recent advances in machine learning. For each method, we describe the general goal (e.g., CDR prediction or whole variable region prediction), the accuracy of the most difficult region, the CDR-H3, its code/server availability, and the source paper. Please note that the CDR-H3 root mean square deviations (RMSDs) are not directly comparable as they could have been obtained from a different test set and are sometimes calculated in a different fashion, e.g., based on Cα or main chain heavy atom positions. As a baseline and reference point, we also include the AlphaFold2 predictions since many methods report values with respect to that method.

Method	Problem addressed	Model characteristic	CDR-H3 prediction accuracy	Corresponding AlphaFold2 accuracy	Availability	Source
DeepH3	CDR prediction	Residual neural network	2.2 Å backbone atoms are used	N/a	https://github.com/Graylab/deepH3-distances-orientations	Ruffolo et al. (2020)
Quaternion and Euler angle combined method	CDR prediction	Graph neural network	SAbDab benchmark: 2.29 Å	N/a	N/a	Son et al. (2022)
ABlooper	CDR prediction	Graph neural network based	RosettaAntibody benchmark: 2.49 Å; SAbDab latest structures: 2.72 Å. Backbone atoms were used	RosettaAntibody benchmark: 2.87 Å	https://github.com/oxpig/ABlooper	Abanades et al. (2022a)
DeepSCAb	Antibody side chain prediction	Residual neural network	Not applicable (side chain prediction)	N/a	https://github.com/Graylab/DeepSCAb	Akpinaroglu et al. (2022)
NanoNet	Heavy chain prediction	Residual network	RosettaAntibody benchmark: 2.38 Å; Nanobodies: 3.16 Å. Backbone atoms were used	Nanobodies: 2.88 Å	https://github.com/dina-lab3D/NanoNet	Cohen et al. (2022)
AbodyBuilder2	Variable region prediction	Based on AlphaFold2 structural module	AbodyBuilder2 benchmark: 2.81 Å. Backbone atoms were used	AbodyBuilder2 benchmark: 2.90 Å	https://github.com/oxpig/ImmuneBuilder	Abanades et al. (2022b)
EquiFold	Variable region prediction	SE(3)-equivariant neural network	IgFold benchmark: 2.86 Å (only N, Cα, C, and O RMSD)	IgFold benchmark: 3.02 Å	https://github.com/Genentech/equifold	Lee et al. (2022)
tfold-Ab	Variable region prediction	Based on AlphaFold2, using language models in the place of Evoformer	IgFold benchmark: 2.74 Å; SAbDab-22H1-Ab benchmark: 3.03 Å. Backbone atoms were used	IgFold benchmark: 3.02 Å; SAbDab-22H1-Ab benchmark: 3.18 Å	https://drug.ai.tencent.com/en	Wu et al. (2022)
xTrimoABfold	Variable region prediction	Based on AlphaFold2, using language models in place of Evoformer	1.25 Å (Cα only)	1.47 Å	N/a	Wang et al. (2022)
IgFold	Variable region prediction	Graph transformer using language model AntiBERTy	IgFold benchmark: 2.99 Å (backbone heavy atoms)	IgFold benchmark: 3.02Å	https://github.com/Graylab/IgFold	Ruffolo et al. (2022a)
AbFold	Variable region prediction	Based on AlphaFold2	AbFold benchmark: 3.04 Å, (backbone heavy atoms)	AbFold benchmark: 3.14 Å (backbone heavy atoms)	N/a	Peng et al. (2023)
AbBERT-HMPN	Sequence and structure generation	Deep graph neural network employing language models with generative capabilities	2.38 Å backbone atoms were used	N/a	N/a	Gao et al. (2022)
RefineGNN	CDR prediction and design	Graph neural network with generative capabilities	2.50 Å (Cα only)	N/a	https://github.com/wengong-jin/RefineGNN	Jin et al. (2021)
AbDockGen	CDR-H3 prediction, design, and antigen docking	Graph neural network-based with generative capabilities	Not applicable (docking scores reported)	N/a	https://github.com/wengong-jin/abdockgen	Jin et al. (2022)
DiffAb	Antibody sequence and the structure design	Diffusion method	Test set of 19 complexes: 3.246 Å (Cα only)	N/a	https://github.com/luost26/diffab	Luo et al. (2022)
DeepAb	Variable region prediction	Residual neural network	RosettaAntibody benchmark: 2.33 Å; therapeutics: 2.52 Å. Backbone heavy atoms were used	N/a	https://github.com/RosettaCommons/DeepAb	Ruffolo et al. (2022b)

## 3 Current machine learning methods tackling antibody structure prediction

### 3.1 What data fuel the models?

Antibody-based deep learning methods require antibody structures for training and validation which are typically downloaded from the Protein Data Bank (PDB). At the time of writing, there were approximately 7,000 redundant antibody structures. Although this sample of the antibody sequence space represents a small subset of all possible antibody molecules (>10^15^), it can still be used to model most naturally occurring antibodies (Krawczyk et al., 2018).

Databases such as AbDb (Ferdous and Martin, 2018) and SAbDab (Dunbar et al., 2014) curate such antibody-specific information. Most of the antibody structure prediction tools use these two resources that facilitate the creation of training, validation, and test datasets.

### 3.2 How is the antibody model quality assessed?

In the original AlphaFold2 work and CASP competition in general, the structural accuracy is calculated using GDT_TS (Kryshtafovych et al., 2021). This score is a measure of structural alignment between the model and native structure that is capable of indicating fold similarities. All antibodies are already of the same fold and one needs to account for differences in single loops (e.g., CDR-H3), where the RMSD is more suitable.

Methods that attempt the modeling of the entire variable region report the entire chain RMSD, further dividing it into the individual CDR RMSDs. Nevertheless, here, the gains in structure prediction accuracy are typically small as such predictions are already of very good quality, excluding the CDR-H3.

Since the CDR-H3 is the most difficult to predict, it is the benchmark point of reference for accuracy across different models. Methods typically report the RMSD of the prediction versus the native structure. RMSD can be calculated using two different approaches. Typically, RMSD is calculated based on the backbone atoms (N, C, CA, and O) or C-alpha (Cα) carbons only, with the latter always being lower. RMSD can also be calculated after aligning the entire variable region or only after aligning the CDR-H3 atoms. The latter method leads to a slightly lower reported RMSD, as it causes bias in the structural alignment for a better fit.

### 3.3 What methods and techniques are used for modeling individual antibody loops and individual chains?

Due to the importance of the CDR-H_3_ loop, many methods focus exclusively on modeling this region. For instance, DeepH_3_ and ABlooper were designed for CDR-H_3_ loop prediction, rather than addressing the entire variable region. DeepH_3_ is based on a residual network architecture that receives one-hot encoding of the sequence to be predicted as input. In terms of residual network size, it is much smaller than RaptorX (Källberg et al., 2012) on which it is based (6 1D + 60 2D) with only 3 1D + 25 2D blocks. It operates by predicting discretized inter-residue distances (assigning distances into equally spaced intervals) and orientation angles which are employed for full structure reconstruction by RosettaAntibody. In contrast, ABlooper is based on equivariant graph neural networks [E(G)NNs], which are equivariant to translations and rotations in 3D space (Satorras et al., 2021). ABlooper allows for coordinate uncertainty estimation by calculating the agreement between five independently trained neural network models. The chief advantage of ABlooper is speed, as it can produce hundreds of structures within seconds as opposed to previously available homology modeling methods that required around a minute.

Beyond CDR-H_3_-focused predictions, one needs to contextualize this loop to the rest of the heavy chain. One of the early machine learning models that could perform whole chain predictions is NanoNet. Originally designed as a predictor of single-chain antibodies (Deszyński et al., 2022), it can also predict heavy chains of canonical antibodies. Similar to DeepH_3_, it is a residual neural network (ResNet) that relies on one-dimensional convolutions to map sequence elements to three-dimensional coordinates. Unlike DeepH_3_, which operates on invariant features (residue distances and orientation angles), NanoNet operates on a single frame of reference by aligning all PDB heavy chains to a single reference structure. Owing to this, the predictions of the NanoNet are 3D coordinates, not requiring further translation into the structure as is the case with invariant features. In the context of the entire heavy chain prediction, authors report 2.38 Å accuracy for CDR-H_3_ (solutions in the region of 1 Å can be considered near-native). Similar to ABlooper, NanoNet is rapid, allowing for predicting thousands of structures in a matter of seconds. However, the predicted structures are often of bad physical quality [e.g., atomic clashes, D-amino acids, etc. (Fernández-Quintero et al., 2023)], requiring refinement.

### 3.4 What architectures and techniques are currently used to predict the entire antibody variable region structure?

Prediction of the entire variable region requires modeling and multimeric assembly of both heavy and light chains. Herein, DeepAb is a network that predicts discretized residue distances and orientation angle bins that are then passed for structure realization using Rosetta. The chief innovation of DeepAb is the usage of a language model as an input to the network. Employing embedding (internal efficient representations of input antibody sequences) for prediction offers an opportunity for the network to perform prediction on more efficient features extracted by the language model. Furthermore, the network employs attention mechanisms that allow tracking of which residues contribute to each other’s predictive signal.

Residual neural networks provide limited ways to abstract invariant three-dimensional information. Representing the entire variable region structure as a graph (as was the case with ABlooper) offers a solution to this problem. For instance, one can encode amino acids as nodes (using features such as amino acid and position) and draw edges between nodes/residues in proximity (e.g., within 8 Å heavy atom distance). Graph neural networks (GNNs) have increasingly gained popularity; this is hallmarked by ABlooper, IgFold, and EquiFold. The authors of EquiFold employed a coarse-grained representation for nodes to demonstrate its power within the framework of a SE (3) (special Euclidean (3) group ensuring rotation and translation equivariance) equivariant network. Ensuring geometric equivariance helps the network in learning features that can be rotated and translated. A more abstract representation using quaternions and Euler angles to encode the amino acids as invariant representations and as an extension of RefineGNN residues has been shown to achieve CDR-H3 predictions in the region of 2.5 Å. IgFold is another GNN-based method that also employs embeddings from AntiBERTy, which is trained on 500-m antibody sequences to supplement its prediction of the entire variable region.

Three key components have contributed to the success of AlphaFold2: the Evoformer, invariant point attention (IPA), and recycling. First, AlphaFold2 infers spatial constraints between amino acids by extracting evolutionary information embedded within multiple sequence alignments (MSAs) using its Evoformer module. In parallel, this information is fed into a structural module that leverages IPA to predict coordinates. IPA is a novel attention mechanism designed to be invariant to rotations and translations by aligning the feature vectors based on the geometric relationship between the residues without changing their 3D positions. It has been shown that it improves the accuracy of protein structure prediction by enabling the network to better capture the complex spatial relationships between residues in a protein. Finally, the whole workflow is repeated or “recycled” three times to refine the prediction.

While AlphaFold2 was designed for predicting any arbitrary protein sequence, its main components have influenced the design of antibody-specific tools. There are variations in the implementation of each of the aforementioned three components. For example, IgFold uses separated weights for each IPA layer and gradient propagation through rotations. xTrimoFoldAb and tfold-Ab use language model embeddings to replace the Evoformer, before applying the learned constraints into the structural module. Other methods, such as ABodyBuilder2, demonstrated that one can use only the structural module without resorting to antibody-specific embeddings or modified Evoformers. The antibody-focused methods are more accurate than AlphaFold2, but these improvements are limited. One major advantage of antibody-specific methods is their efficient running time. For instance, ABodyBuilder2 achieves predictions in a matter of seconds, compared to tens of minutes for AlphaFold2. AlphaFold2’s running time is comparatively long because of the MSA search step, which is unnecessary for antibody-specific methods.

The loss function drives the training of a model as it penalizes wrong predictions and rewards better ones. It is extremely important as one of the chief innovations of AlphaFold2 was the introduction of the frame aligned point error (FAPE) loss. This component exposes the model to information related to physicochemical constraints, such as proper chemical bond distances and angles, as well as penalizing atom clashes and other structural violations, and is also used in some of the antibody-specific models. However, because of the skewed difficulty in structure prediction, applying the same loss to each antibody region is not an ideal approach. For instance, xTrimoABFold employs focal loss focused on CDRs that are more difficult to predict. On the other hand, ABodyBuilder2 treats framework and CDR regions differently, clamping framework regions at a FAPE loss of 30 Å and the CDRs’ FAPE loss at 10 Å.

### 3.5 How do networks approach fine-structural details beyond the backbone?

Despite the progress in predictions, a seemingly trivial problem faced by the networks is the physical plausibility of the produced models (Fernández-Quintero et al., 2022). It was observed in AlphaFold2 that the structure module can produce predictions violating physical constraints, such as atomic clashes. This is not only a problem of AlphaFold2-based methods, and methods such as NanoNet and EquiFold are also afflicted. Methods such as ABodyBuilder2 and IgFold employed OpenMM and Rosetta, respectively, to reduce the number of physical clashes in the model produced by a neural network. The number of non-physical distances can also be reduced by introducing various physical constraints at the training time (Eguchi et al., 2022; Kończak et al., 2022).

Although structure prediction is typically evaluated on its ability to recapitulate the backbone, the determination of the side chains is important for fine-grained modeling of molecular function, such as binding affinity. Methods such as ABodyBuilder2 and IgFold produce the backbone structures annotated with side chains. Other methods such as EquiFold use a novel coarse-graining scheme where atoms are mapped to coarse-grained “superatom” prior to structural modeling and then reverse-mapped to the individual atoms once the backbone is constructed (Akpinaroglu et al., 2022). Other methods such as NanoNet only produce the backbone. Side chains are typically added by algorithms such as SCWRL (Krivov et al., 2009) or PEARS (Leem et al., 2018), but recently an antibody-specific side chain prediction mechanism using convolutional neural networks has been introduced—DeepSCAb (Akpinaroglu et al., 2022). Altogether, fast and accurate prediction of all-atom models is key to using the antibody structures for practical drug discovery purposes.

## 4 Drug discovery perspective of antibody structure prediction

Antibodies are a well-established drug format, with the structure as a key component in aiding their discovery and development, paving ground for real-world applications of 3D modeling.

Antibody structures provide rich information that can be used to improve various prediction features, such as molecular recognition (Oh et al., 2021), liability detection (Irudayanathan et al., 2022), or developability screening (Jain et al., 2023). These models can complement wet-lab antibody discovery methods, such as immunization or phage display, to ultimately improve the selection of binders. For instance, the identification of antigen-specific antibodies was typically tackled using clonotype/sequence clustering methods. Machine learning has shown alternative ways to group these molecules such as by embeddings from variational autoencoders (VAEs) (Friedensohn et al., 2020), predicting paratope residues (Richardson et al., 2021), or clustering structures (Robinson et al., 2021). In particular, structural clustering can provide a highly translational interpretation of antibody binding. The methods described in this Review are highly scalable, making it possible to group thousands of structures.

The optimization of biologics is the process of improving an existing molecule, which already displays a variety of desirable properties, with regard to a set of physicochemical properties. Structural features can be employed to guide the optimization process. A trivial example would be to focus existing liability removal (e.g., deamidation) protocols on only surface-exposed residues, which can be identified based on a reasonably accurate three-dimensional model (Irudayanathan et al., 2022). Structural features can be indicative of successful therapeutics (Raybould et al., 2019; Ahmed et al., 2021), with some differences in the calculated results based on the underlying modeling method (Jain et al., 2023). In some cases, such as antibody–antigen docking, good quality models are needed to reach the results achieved by docking crystal structures (Schneider et al., 2021).

The most ambitious use of antibody structure prediction is for the *de novo* antibody design, where the goal is to computationally define an antibody sequence that can bind to a given target epitope. One approach to the *de novo* design that relies on structural predictions is “virtual screening,” a methodology that has been practiced in small molecule drug discovery for decades but has only been recently applied to antibodies. This can involve the modeling of and selection from millions of antibody molecules, which are then funneled into a molecular docking approach (Schneider et al., 2021; Jin et al., 2022) or alternative binding site design methods (Rangel et al., 2022). The quality of the models is a key consideration as subtle changes in Vh/Vl arrangement, backbone, or side chain orientation can affect the quality of the predictions (Fernández-Quintero et al., 2022). In addition, any such efforts hinge on linking the antibody structural predictions to paratope–epitope interaction prediction. In this context, “zero-shot” predictions require the models to propose sequences binding a specific epitope without observing it, or any close variants of it, in the training/test sets.

Another approach to the *de novo* design is using generative methods. Herein, the latent space of the input (e.g., antibody sequences) is learned, providing a way to sample novel elements. Producing novel sequences based on transformer models has already been shown in general proteins (Rives et al., 2021) as well as in the antibody world (Melnyk et al., 2021; Saka et al., 2021; Shin et al., 2021; Shuai et al., 2021). Autoregressive methods such as IgLM (Shuai et al., 2021) offer a way to generate new binder sequences based on millions of sequences from natural repertoires. Such generation can also be biased toward sequences with certain biophysical properties by GANs (Amimeur et al., 2020). Most such methods, however, are currently sequence-driven but not structure-driven.

Structure holds the potential to provide more information than sequence alone (Kovaltsuk et al., 2017). Encoding the structural space, in the form of torsional angles using VAEs, has shown potential in generating novel 3D shapes (Eguchi et al., 2022). Leveraging structural information for generating paratopes to specific antigens should produce better results than using sequence alone (Jin et al., 2022). Higher quality structural models have the potential to inform better structure-generation methods, leading to more accurate emulation of molecular space than sequence alone. Embeddings generated by the inverse-folding of general proteins have already shown potential to be useful for B-cell epitope prediction (Hsu et al., 2022; Høie et al., 2023).

In the context of structure-conditioned generative methods, RefineGNN, AbDockGen, AbBERT-HMPN, and DiffAb go a step further than the modeling methods described in this review. They also provide a “compatibility” score for the structure and designed sequence. RefineGNN, AbDockGen, and AbBERT-HMPN are based on the iterative refinement of latent representations from graph neural networks, whereas DiffAb samples via a denoising diffusion model. The integration of structure prediction and sequence design is the next intuitive step superseding structure prediction, which holds the promise to enhance antibody-based drug discovery.

## 5 Conclusion

Advances in protein structure prediction have practical application in the discovery of new antibody drugs.

In general, accuracy increasing with respect to the pioneer in ML-based accurate structure prediction, AlphaFold2, is noticeable, but stay within an order of magnitude. Predictions of the CDR-H3 structure in particular appear to be “stuck” in the 2–3 Å heavy atom backbone RMSD interval. Difficulty in the prediction of CDR-H3 conformation could stem from the loops’ flexibility (Wong et al., 2011; Fernández-Quintero et al., 2018; Jeliazkov et al., 2018) as well as the possible influence of the Vh/Vl orientation (Marze et al., 2016; Boucher et al., 2023). With only several thousand antibody structures at hand (Dunbar et al., 2014), it is challenging to study any flexibility or allosteric effects, but perhaps with a larger number of better quality cryo-EM structures we will increase the volume of structural information available. Efforts in improving antibody structure prediction might take the flexibility into account by scoring the CDR-H3 conformational ensemble rather than single “best structure” produced.

The main advantage of the antibody-specific methods with respect to AlphaFold2 is the speed. The antibody sequence space in a single individual [∼10^9^–10^11^ (Briney et al., 2019)] easily surpasses the human proteome (∼20 k). The speed of antibody modeling methods is of utmost importance, as it directly translates to the mapping of the available antibody sequence space (Kovaltsuk et al., 2018; Olsen et al., 2022), antibody virtual screening (Schneider et al., 2021; Rangel et al., 2022), and the development of novel generative models (Eguchi et al., 2022).

Given the number of currently available antibody-specific structure predictions, it might be suitable to take stock of the state of the field and devote efforts into benchmarking the different methods as was the case with the two rounds of the Antibody Modeling Assessment competition (Almagro et al., 2011; Almagro et al., 2014). In the field of antibody discovery specifically, we could use the tools not only to test by a single measure of RMSD but also to assess how useful the structural predictions are for therapeutically minded tasks, such as lead optimization, docking, epitope, or paratope prediction.

Altogether the accuracy, speed, and accessibility of the current antibody modeling methods make it possible to apply structural information to various aspects of biologics discovery pipelines today. An incremental improvement to existing discovery approaches using structure-guided computational methods appears entirely feasible, while the field continues to move ever forward toward the “holy grail” of the true *de novo* antibody design.
